# Health-related quality of life after major extremity trauma: qualitative research with military service members and clinicians to inform measurement of patient-reported outcomes

**DOI:** 10.1007/s11136-025-03915-0

**Published:** 2025-02-20

**Authors:** Callie E. Tyner, Pamela A. Kisala, Jerry Slotkin, Matthew L. Cohen, Jill M. Cancio, Alison L. Pruziner, Christopher L. Dearth, David S. Tulsky

**Affiliations:** 1 Center for Health Assessment Research and Translation, University of Delaware, Newark, DE USA; 2 Department of Communication Sciences & Disorders, University of Delaware, Newark, DE USA; 3 US Army Institute of Surgical Research, JBSA Fort, Sam Houston, TX USA; 4 Extremity Trauma and Amputation Center of Excellence, Defense Health Agency, Falls Church, VA USA; 5 Walter Reed National Military Medical Center, Bethesda, MD USA; 6 Department of Veterans Affairs, National Veterans Sports Programs and Special Events, Washington, DC USA; 7 Uniformed Services University of the Health Sciences, Bethesda, MD USA; 8 Departments of Physical Therapy and Psychological & Brain Sciences, University of Delaware, Newark, DE USA

**Keywords:** Patient reported outcome measures, Quality of life, Focus groups, Qualitative research, Amputation, surgical

## Abstract

**Purpose:**

The purpose of this study was to understand health-related quality of life (HRQOL) factors for adults who experience major extremity trauma, including limb preservation and amputation, to guide the selection and creation of patient-reported outcome (PRO) measures.

**Methods:**

A thematic content qualitative analysis was used to study service members (SMs) with a history of major extremity trauma and clinical providers with expertise in limb trauma, limb loss, and limb preservation/reconstruction. Focus groups were conducted at three Military Treatment Facilities and one Department of Veterans Affairs Medical Center.

**Results:**

Fifty-six SMs and 34 clinicians participated. Thirty-six percent of focus group comments were coded under Physical Health, 31% Emotional Health, and 28% Social Participation. These results were largely consistent across clinicians and SMs, and clinical subgroups, with a few exceptions such as the relevance of fine motor tasks and prosthetic devices for SMs with upper extremity injury/limb loss, and orthotic devices for SMs with limb preservation/reconstruction.

**Conclusion:**

Many HRQOL topics identified are shared with existing general PRO measures—including pain, physical function, anxiety, depression, anger, positive affect and well-being, fatigue, social participation, and loneliness—as well as rehabilitation-focused PRO measures—such as resilience, grief/loss, stigma, self-esteem, mobility, fine motor functioning, self-care, and independence. This qualitative research can be used to inform domains of HRQOL in need of new PRO measures for this population, including satisfaction with orthosis/prosthesis, satisfaction with physical abilities/athleticism, body image, future outlook, and vocational impact.

**Supplementary Information:**

The online version contains supplementary material available at 10.1007/s11136-025-03915-0.

Major extremity trauma represents a significant cause of disability among U.S. military service members (SM), both on and off the battlefield. After two decades of armed conflicts in Afghanistan and Iraq, injuries to the extremities have accounted for approximately half of all battlefield injuries [1]. It has been estimated that 5,684 major limb injuries occurred as a result of these conflicts, including approximately 1700 individuals with battle-injury-related major limb loss [2, 3]. These high numbers have been attributed to the unprecedented rate of explosive-related combat casualties, ranging from 74 to 88% of casualties experienced among SMs [3–5]. Extremity injuries are also the most common type of *non-combat* traumatic injury for SMs *and* civilians, representing over 60% of injuries, with more than 20,000 new extremity injuries occurring every year in the U.S [6].

The rehabilitation needs of this population are heterogeneous and complex, and injury severity alone does not adequately predict long-term disability or return to duty for SMs [7]. Instead, an individual’s experience of post-injury disability reflects a confluence of factors that include physical functioning (e.g., medical comorbidities [8–12], activities of daily living [13, 14], and use of adaptive technologies [11, 13]), emotional experiences (e.g., depression, anxiety, trauma, grief, and self-esteem [15–18]), and psychosocial difficulties (e.g., occupational changes and retirement from active duty military service [13, 15]). For example, only 2% of SMs with limb loss return to their original occupations [7]. Because of this, many injured SMs are faced with the challenges of reintegration into civilian life, complicated by physical limitations and changed self-image. Health-related quality of life (HRQOL) is a construct that encapsulates how physical, emotional, and psychosocial challenges affect an individual [19]. Patient-reported outcome (PRO) measures of HRQOL provide useful tools for measuring outcomes and evaluating interventions [20]. Integration of PRO measures in clinical care has also been shown to improve healthcare, specifically in the domains of patient-provider communication, patient satisfaction, monitoring of treatment response, and detection of unrecognized problems [21].

Over the last 20 years, there have been several national and international initiatives to develop PROs. The Patient Reported Outcomes Measurement Information System (PROMIS^®^) is a comprehensive system of generic item banks that measure mental, physical, and social health [22]. The National Institutes of Health (NIH) Toolbox^®^ Emotion Battery is a set of generic item banks of positive and negative emotions, social relationships, stress and self-efficacy [23]. Similarly, the Quality of Life in Neurological Disorders (Neuro-QoL) complements the PROs available in PROMIS and the NIH Toolbox with the development of similar item banks, but tailored to individuals with neurologic disorders, specifically stroke, Parkinson’s disease, multiple sclerosis, epilepsy, amyotrophic lateral sclerosis, and muscular dystrophy [24–26]. Additional measurement systems have been developed to focus on individuals who have had sudden, traumatic injuries (e.g., Spinal Cord Injury-Quality of Life [SCI-QOL]; Traumatic Brain Injury-Quality of Life [TBI-QOL]; and Spinal Cord Injury-Functional Index [SCI-FI]), which have identified targeted areas of functioning unique to individuals with SCI or TBI as well as optimizing the existing scales from PROMIS and Neuro-QoL [27–31]. Qualitative research is typically used to guide the identification of issues for these comprehensive measurement systems. However, major extremity trauma and limb loss have not been a focus of these systems, and the measures have not been validated for use in this population.

A great deal of research with this population has used generic scales of QOL (e.g., SF-36, Sickness Impact Profile), which are primarily used as general/overall ratings of outcomes and can be difficult to interpret because physical, functional, and psychosocial symptoms are often blended into one score [32, 33]. PRO measures that measure physical, emotional and social functioning independently have grown in popularity in orthopedics, particularly the PROMIS measures [34, 35]. There are some high-quality condition-specific PRO instruments that have been designed and/or used for evaluation of outcomes for individuals with limb loss, such as the Orthotics Prosthetics Users Survey (OPUS) [36], the Prosthetic Limb Users Survey of Mobility (PLUS-M) [37, 38], or the Patient Experience Measure (PEM) [39]. However, these measures are only for device users, or are specific to individuals with either upper- or lower-limb loss only (PEM and PLUS-M, respectively). Further, the OPUS and PLUS-M were not designed specifically for individuals with traumatic limb loss, which is experientially quite different from the more common causes of lower-limb loss, specifically diabetes mellitus, peripheral vascular disease, and neuropathy [40].

Therefore, the present study was designed to document the HRQOL considerations that are relevant to SMs who have experienced major extremity trauma, regardless of limb or device-use status. While the research team took a constructivist perspective on individual experiences of HRQOL, we simultaneously approached this research with post-positivist assumptions about the ability of qualitative researchers to discover a true, shared understanding of HRQOL that is applicable across a wide variety of and social and injury-related factors and individual lived experiences [41]. The immediate goal of this project was to identify and describe the most important domains of functioning and factors relevant to HRQOL for understanding the rehabilitation needs of this population. The ultimate goal of this work is to develop a PRO measurement system to better assess and, ultimately, improve HRQOL outcomes in individuals who have sustained severe limb trauma.

## Methods

This study employed a well-established methodology, as described by Kisala and Tulsky [42], for identifying important HRQOL topics for rehabilitation populations, which utilizes open-ended and free-flowing discussion of the experiences of individuals in the target population. This method places the person with the disability and their clinicians in the role of expert in determining the most important HRQOL issues to measure. The goal of this approach is to validate and optimize existing PRO measures—and supplement with new measures if needed—so that a comprehensive assessment system could be tailored to this population. This research methodology follows the PRO measure development guidelines promoted by the NIH PROMIS working group [43, 44] and the ISOQOL recommendations for patient-centered outcomes [20].

### Participants and focus group design

Focus group participants were recruited from three Military Treatment Facilities and one Department of Veterans Affairs (VA) medical center. Participants included SMs with limb loss and/or preservation/reconstruction and clinical providers with expertise in limb trauma and loss. All research activities were IRB-approved. Two moderators were present for all focus groups to introduce the activity, encourage participation from each group member, and provide an organized structure to the discussion. The moderators’ primary goal was to foster a spontaneous discussion, with limited prompting or directing. The moderators were interested in covering most major domains of HRQOL (physical, emotional, and social), but did not press if a domain did not generate much conversation. Each focus group lasted between 60 and 90 min. This methodology parallels that used in the development of PROMIS and Neuro-QoL instruments [45] and was selected to capture stakeholder feedback as thoroughly as possible while attempting to minimize the effects of researcher bias. See the supplementary material for additional details.

### Qualitative analysis

Audio recordings of the focus groups were transcribed and reviewed by the study team for accuracy before beginning qualitative analyses. A thematic content analysis was used [46, 47]; many details of our methodology have been described in previous work [29, 42, 48–50] and only a brief overview will be provided here. Notably, while our methods were originally developed from a grounded theory perspective [51, 52], they have evolved over time to contain details not endorsed by grounded theory, and thus our conceptualization has therefore evolved to fit within a post-positivist thematic analysis approach. We also perform quantification (a component of content analysis [42]) as a final analysis step to determine the relative frequency of mention of each theme or code [53].

First, two investigators reviewed all transcripts carefully and developed a preliminary list of domains and subdomains based upon the issues discussed during the focus groups. This initial *open coding* task focused on the enumeration of all potentially relevant concepts that were discussed during the focus groups. The content was then re-reviewed for two purposes. First, it was imperative to harmonize nomenclature not only across the two investigators but also with terms commonly used in PRO measurement, to facilitate determination of coverage of key concepts by existing PRO measurement systems. Next, the investigators used *axial coding* to identify the interrelationships between concepts and restructured the codes into a *codebook*—that is, a hierarchical structure of codes accompanied by definitions and inclusion/exclusion guidelines [54]. The codebook was then reviewed by multiple investigators. Code definitions were developed or expanded, and examples were added as appropriate.

*Selective coding* is the process of integrating and refining the concepts constructed during analysis [p. 143; 52]. In an effort to find the most central themes which should be represented in a PRO measurement system for individuals with limb trauma, we implemented a procedure in which two or more raters applied codes from the codebook to all relevant segments of transcript text. Research has shown that it is important to ensure that independent coders are trained and provided enough information to interpret the codebook consistently [55]. Hence, as a first step in this process, the open-ended feedback provided during the focus groups had to be organized into statements to which the coders could assign distinct values. Each of the transcripts (one per focus group) was parsed into discrete segments of text or *chunks* that communicated a single idea. Next, four research assistants were trained as independent raters on the codebook content and completed an inter-rater reliability exercise to ensure that they achieved at least 80% consistency before proceeding. All coding was performed using Nvivo software [56]. Two raters coded every chunk within each transcript according to the codebook. After coding a complete transcript, the two raters met to compare and review any conflicting codes. If the coders were unable to come to a consensus, then a supervising investigator (author PK) would intervene to code the ambiguous chunks.

Last, descriptive statistics were computed for the frequency that the codes appeared across the corpus of qualitative data. Descriptive statistics were also explored by considering frequency of mention of the codes by participant type (SM vs. clinician), injury location (upper extremity vs. lower extremity vs. both), and treatment (limb loss vs. limb preservation/reconstruction). The final results demonstrate the frequency with which each code was applied, thereby suggesting the codes’ relative importance in this population [42, 57, 58].

### Trustworthiness of qualitative data/analysis

The trustworthiness of the qualitative data collection and analysis was assured through its credibility (e.g., through data triangulation from multiple groups and participant types), transferability (e.g., likely applicability of findings to other individuals with limb trauma, given the number of participants and groups and the variety of participant types), dependability (e.g., standardized and well-documented methods for data collection and analysis), and confirmability (e.g., through data triangulation and use of a clearly documented coding schema).

## Results

### Participants

Participants included 56 SMs and 34 clinicians. Most SMs were male (*n* = 50). See Table 1 for demographic and clinical characteristics of the SMs. The modal clinical provider specialties were physical therapists and prosthetists/orthotists. Most clinicians were male (*n* = 23). See Table 2 for their professional and demographic characteristics.

**Table 1:** Demographic and clinical characteristics of SMs in focus groups (*n* = 56)

Variable	M (SD)	median (range)
Age (years)^*^	35.8 (9.52)	34 (22–64)
Time since injury (months) ^**^	49.7 (46.36)	30 (3-196)
**Limb Injury Location**	**N (%)**	
Upper extremity limb loss	11 (19.6)	
Lower extremity limb loss	16 (28.6)	
Limb preservation/reconstruction	20 (35.7)	
One or more limb loss and/or surgery	9 (16.1)	
**Race**	**N (%)**	
White	32 (57.1)	
Non-White^***^	9 (16.1)	
Unknown	14 (25.0)	
Do not wish to provide	1 (1.8)	
**Ethnicity**	**N (%)**	
Hispanic/Latino	9 (16.1)	
Non-Hispanic/Latino	33 (58.9)	
Do not wish to provide	1 (1.8)	
Unknown	13 (23.2)	

Note: ^*^Age missing from 11 SMs; ^**^Time since injury missing from 6 SMs; ^***^Includes Asian and Black or African American (categories collapsed due to low frequencies to avoid statistical disclosure)

**Table 2:** Professional discipline, years practicing, age, race, and ethnicity of clinicians in focus groups (*n* = 34)

Professional Discipline	*N* (%)
Kinesiotherapist	1 (2.9)
Nurse	2 (5.9)
Occupational Therapist	5 (14.7)
Physiatrist	6 (17.7)
Physical Therapist	8 (23.5)
Prosthetist and/or Orthotist	8 (23.5)
Psychologist	2 (5.9)
Recreational Therapist	2 (5.9)
**Age & Experience** ^*^	**M (SD)**
Provider Age	42.9 (11.6)
Years Practicing	15.9 (11.0)
**Provider Race**	**N (%)**
White	14 (41.2)
Non-White^**^	4 (11.8)
Unknown	16 (47.1)
**Provider Ethnicity**	**N (%)**
Hispanic/Latino	1 (2.9)
Non-Hispanic/Latino	15 (44.1)
Do not wish to provide	2 (5.9)
Unknown	16 (47.1)

Note: ^*^Information missing from 16 participants; ^**^Includes American Indian or Alaska Native, Asian, and Black or African American (categories collapsed due to low frequencies to avoid statistical disclosure)

### Groups

Of the 20 total sessions, 16 were with SMs and four with clinicians. The sessions with SMs ranged in size from one to seven participants (mean = 3.5, SD = 1.75); one session included only one participant due to scheduling challenges. Of the SM groups, seven included participants with lower extremity injuries only, three included individuals with upper extremity injuries only, and six included individuals with upper and lower injuries (both within and across individuals). Eight of the SM groups were limited to participants with limb loss only, two with limb preservation only, and six groups included individuals with both limb loss and limb preservation. The focus groups with clinicians ranged in size from eight to 10 participants (mean = 8.5; SD = 1.0).

### Qualitative results and relative frequencies

The final codebook included 95 codes (four parent, 24 child, 67 grandchild or smaller), with the domains of Physical Health (36% of comments), Emotional Health (31% of comments), Social Participation (28% of comments), and Cognition (< 1% of comments) representing the four parent codes. After data were coded, we reviewed the applied codes in order by group to determine if and when saturation of themes (that is, the point at which new themes no longer identified from data) had occurred [44, 45]. We determined that 84% of codes were identified by three sessions, 97.9% by seven sessions, and 100% by 10 sessions. Since we conducted several subsequent focus groups with a variety of subgroups (upper and lower, limb loss and preservation, clinicians and patients), we were confident that saturation of themes had been reached, and no further groups were needed. In the following sections, we present the relative frequencies of child/grandchild codes as a percentage of each parent code. In the Supplementary Material (Tables S1, S2, and S3), results are shown for the overall sample. In these supplementary tables, results are also broken down by the SMs vs. clinicians, and clinical subgroups of injury location and treatment type.

#### Physical health

36% of all focus group comments were related to the parent code Physical Health, which includes child codes related to both functioning and symptoms. Medical Health/Issues was the largest child code (27% of Physical Health comments), see supplementary material for information on grandchild and great-grandchild codes. Other frequently applied Physical Health child codes included Mobility (16%), Orthosis/Prosthesis (15%), Pain (12%), Satisfaction with Physical Abilities/Athleticism (11%), Medication (10%), and Upper Extremity Function/Self-Care (6%). Results for all Physical Health child codes are provided in Table S1.

Delving deeper into the Physical Health codes, we observed that Pain was connected with many aspects of adjusting to daily life after an injury. One example was learning how to pace oneself in light of new limitations: “*I battle that happy medium where my doctor says*,* ‘Don’t do too much.’ And I’m like*,* ‘What is too much?’ He was like*,* ‘Well anything that you do that causes your leg to hurt for days after is too much.’ And I said*,* ‘Everything I do*,* every therapy session I go to*,* causes me pain that lasts for more than one day. So*,* are you telling me not to do anything?’”* For many participants, comments coded as Sleep and Medication were associated with Pain, for example: *“So*,* we try to sleep*,* and we can’t do that without medications*,* and we’re on so many other drugs. So*,* you know*,* here we are trying to deal with the pain*,* and we’re trying to sleep to get through the pain*,* but without adding another pain medicine*,* we can’t sleep through the night.”* Satisfaction with Physical Abilities/Athleticism was notable in that many of the SMs had high levels of physical fitness prior to their injury, and described using exercise as a way to cope with stress both prior to and after their injury. For example, one participant said: *“But when you’re fighting stuff like depression and everything else*,* exercise is a natural releaser of endorphins*,* makes you feel good about yourself*,* you’re out doing things.”* Some participants transitioned to adaptive fitness after injury and continued to pursue athletics, for example: *“I started adaptive sports*,* because I realized I can’t do what I used to do. But adaptive sports is my new normal*,* like wheelchair basketball*,* seated volleyball*,* hand cycling… That’s what helps me get through.”* Other participants expressed more intense dissatisfaction with their physical limitations and decreased physical fitness, particularly among individuals who described athleticism as an important part of their pre-injury identity. More quotes from the Physical Health codes appear in Table 3.

**Table 3:** Exemplar quotes coded under the physical health parent code

Code	Exemplar Quote
Medical Health/Issues	“Being healthy is another thing, because now you lost part of your limbs… you have to think about things like that. Now what am I doing… to keep a healthy heart?…I can’t run now. I’m not weight bearing. Now I’m losing bone density because I’m in a wheelchair… It is [self-perpetuating].” *– Speaker from group #4*,* UE Limb Loss group from Bethesda*
Mobility	“When somebody tells you, you can’t do something, it motivated me to say, ‘Well, yes, I can.’ Only to be humbled by my flesh, that I can’t. I tried things that I was not able to do, hurt myself, set myself back in the healing process because I was so adamant on, I’m going to walk. I’m going to drive. I’m going to ride a bike. And I’m going to run, and I’m going to do this, this and that.” *– Speaker from group #7*,* Limb Preservation group from Bethesda*
Prosthesis	“I’ve found myself to be a different person without my prosthetic… I’m a very confident person, but my prosthetic, it completes me.” *– Speaker from group #4*,* (UE) Limb Loss group from Bethesda*
Orthosis	“I am interdependent with the brace. I want the bracing systems to make me better at what I do. I don’t want to be 100% dependent, if I don’t have to be.” *– Speaker from group #1*,* LE Preservation group from Bethesda*
Pain	“The pain comes in all different forms. There’s a lot of nerve damage, a lot of leg twitching, a lot of pins and needles throughout the whole entire limb from the knee down on my right leg, and there’s the physical pain, too, of actually the weight bearing.” *– Speaker from group #8*,* LE group (comprised of individuals with limb loss and/or limb preservation) from San Antonio*
Satisfaction with Physical Abilities/Athleticism	“I was a long distance runner before I got hurt and I was very active… It was a big loss. Even though it’s been seven years, I still dream about running.” *– Speaker from group #1*,* LE Preservation group from Bethesda*
Medication	“The only reason I have boycotted taking my medications, because it has debilitated me to having accidents, falling, because I’m loopy, not being able to remember conversations, not being able to be independent and make choices, not being able to drive. So, I’ve traded off the least amount of medication that I can take to make it through therapy.” *– Speaker from group #7*,* Limb Preservation group from Bethesda*
Upper Extremity Function/ Self Care	“It’s a big change from being able to, ‘got to go, got to go, I need to take a shower, be in and out in ten minutes, dressed and everything.’ It’s not like that no more. It takes a good half an hour, sometimes maybe 45 min just depending on how I feel that day.” *– Speaker from group #8*,* LE group (comprised of individuals with limb loss and/or limb preservation) from San Antonio*
Sleep Disturbance	“When I first got hurt… sleep was terrible. I mean, I’d take medication, and it didn’t work. I still take medication… But even after the medication, it doesn’t keep me asleep. You know, I’ll sleep two or three hours and wake up, and sleep another hour and wake up. So, sleep is a - it’s a very fleeting thing for me.” *– Speaker from group #10*,* UE and LE group (comprised of individuals with limb loss and/or limb preservation) from San Antonio*
Fatigue	“Can I keep going on? I’m just - my body just feels fatigued. I may get ten hours of sleep, but I’m just fatigued. I just can’t take this.” *– Speaker from group #9*,* Clinician group from San Antonio*
Sexual Health	“One thing that hasn’t been brought out I think is sexual functioning, because obviously a lot of them are also on a chronic level of opioids, which of course is going to affect their testosterone level… I mean, providers get really shy about certain situations, even people who shouldn’t necessarily be shy. Anything from the pain from an actual physical change to just the mechanics of it. You know, what does a sexual relationship look like…? And I think that’s one of the things that’s probably not spoken about that really does affect them.” (Clinician) *– Speaker from group #9*,* Clinician group from San Antonio*

Notes: UE = upper extremity; LE = lower extremity

#### Emotional health

The Emotional Health parent code represented 31% of comments. The most frequent child code was Resilience (33% of Emotional Health comments), defined in this codebook as the ability to adapt to whatever comes one’s way in life and as the subjective experience of adapting to difficult or challenging life experiences, particularly highly stressful or traumatic events [59]. Comments related to motivation, coping, and acceptance were included in this code. Other frequently applied Emotional Health child codes include Anxiety/Fear/PTSD (10%), Anger (9%), Grief/Loss (9%), Future Outlook (8%), Self-Esteem (7%), Stigma (6%), Positive Affect and Well-being (6%), Depression (4%), and Body Image (4%). Results for Emotional Health codes are provided in Table S2.

While many of the concepts coded under Emotional Health, such as Self-Esteem, Depression, and Body Image, are salient to people from many clinical groups, some of the ways that the participants in this study with limb trauma discussed them were unique. For example, the association of Emotional Health with changes in physical function and symptoms: *“Because you’re just constantly in pain*,* you’re in agony*,* you’re not going to be in a good mood. So*,* then you get angry and then you get depressed.”* Body Image changes due to limb loss and other visible signs of injury were salient for many SMs, including this example: *“I had a hard time being like… how can women find me attractive*,* when I look the way I do? Because in the mirror I just see some [expletive] dude missing his legs. I didn’t see me as a person. I just saw the prosthetics.”* Other Emotional Health codes, such as Grief/Loss and Future Outlook, were related to the sudden nature of limb trauma. Participants primarily expressed grief over the loss of their former physical abilities and fitness and, particularly for those who had to discontinue their military careers, their former vocational options. Some SMs described needing to reimagine their life plans, and dealing with unexpected challenges: *“Every day is like a new set of challenges… you don’t really realize how much you won’t be able to do after this injury [until you’ve experienced it firsthand].”* In addition to individuals needing to reframe their own self-appraisals, many also reported feeling stigmatized by others: “*It’s difficult to get out because people always do look at you weird*,* and they always want to come up and ask what happened*,* how it happened*,* and you don’t always want to answer.”* Additional representative quotes appear in Table 4.

**Table 4:** Exemplar quotes coded under the emotional health parent code

Code	Exemplar Quote
Resilience	“I mean the injury itself is not the end of the world. It just gives you another opportunity to find ways to get around things and outthink a problem.” *– Speaker from group #1*,* LE Preservation group from Bethesda*
Anxiety/Fear/PTSD	“We’ve all got PTSD in one way, shape, or form. I don’t care if you got blown across a road, shot in the chest, or had a motorcycle accident. A traumatic experience like that…is going to leave imprints and boot prints in it.” *– Speaker from group #6*,* UE Limb Loss group from Bethesda*
Anger	“I have lots of angry outbursts…I would just take that out in a second on strangers or especially people that you’re close to.” *– Speaker from group #1*,* LE Preservation group from Bethesda*
Grief/Loss	“I used to be able to run around with my nephews, wrestle with them. I can’t do that anymore. Now I feel bad, because I’ll say ‘hi,’ but it’s not anywhere as close as it used to be -- I can’t roughhouse with them like I used to. And that was my way of spending time with them.” *– Speaker from group #16*,* Limb Preservation group from San Diego*
Future Outlook	“You know you wake up in this environment; something happened that brought you here. And that disrupted what you thought - what you had for a plan for the future… You know at different parts along the way there’s a lot of uncertainty as to what the future holds.” *– Speaker from group #1*,* LE Preservation group from Bethesda*
Self-Esteem	“An amputation changes your whole dynamic of how do you view yourself, what your capabilities are, and that feeds into everything else. It can address like self-worth, self-value, how you see yourself in your relationships with… a spouse, friends, family.” *– Speaker from group #12*,* LE Limb Loss group from San Antonio*
Stigma	“So, I think a lot of that is society today places a stigma on people that need help, just because - and especially the military. If you come with that certain approach, you’re considered weak and unfit for duty.” *– Speaker from group #4*,* UE Limb Loss group from Bethesda*
Positive Affect and Well-being	“It’s kind of a mind game, and you have to stay positive and do things that bring you happiness.” *– Speaker from group #4*,* UE Limb Loss group from Bethesda*
Depression	“I was just really like, why get up in the morning? Why keep fighting? You know, I’ve been trying to do this same thing in therapy since day one, and I still can’t do it. Why do they still keep having me try?” *– Speaker from group #7*,* Limb Preservation group from Bethesda*
Body Image	“You know, look at our society today. You open up a magazine and somebody’s gorgeous. You look at our music, and everything. You have those triggers… With my prosthetics, the way clothes don’t fit makes me feel insecure. I can’t wear certain shirts. It’s just - they don’t look good on me. And that’s my opinion of myself, you know.” *– Speaker from group #4*,* UE Limb Loss group from Bethesda*
Health-Related Self Efficacy	“I think teaching people to be their own advocate is a big one… I was supposed to have this appointment. Well, they didn’t call me, okay, so then what are you going to do about it? You going to call?” (Clinician) *– Speaker from group #4*,* UE Limb Loss group from Bethesda*

Notes: UE = upper extremity; LE = lower extremity

#### Social participation

28% of focus group comments fell under the parent code Social Participation, with the majority of comments under the child code Social Relationships (64% of Social Participation comments). Relationships were often described as a source of support: *“I’d say for me it’s brought me and my wife closer. We’re spending more time together because she’s my caregiver.”* Conversely, other relationships were challenging: *“I’ve got friends that are just*,* ‘Why aren’t you better?’… I just want to stay away from them because I know I’m going to get [mad] and probably want to punch them.”*

Other frequently applied child codes of Social Participation include Vocational Impact (18%), Independence (9%), Social Activities (8%), and Loneliness/Social Isolation (2%). Vocational Impact was particularly salient for individuals who had planned on long careers in the military but had to abruptly adjust to life outside the military and consider a civilian career for the first time. Furthermore, changes in career post-injury were often accompanied by lost social connections: “*Your military friends still have their military careers*,* so they pick up where they left off and they move on. And it’s kind of like you’re just left behind. You’re just like*,* all right*,* bye*,* guys*,* like have fun with your life.”* Loneliness was brought up as particularly problematic in the initial months post-injury: “*My first year*,* I was ‘in the dark’ when I got out because I spent most of the time in my house. I didn’t do anything*,* I didn’t want to see anybody… if they wouldn’t say anything about your prosthetic arm*,* they’d look at you*,* and they’d know.”* Results for Social Participation codes are provided in Table S3 and representative quotes appear in Table 5.

**Table 5:** Exemplar quotes coded under the Social participation parent code

Code	Exemplar Quote
Social Relationships	“Having family by your bedside is very important. And I witnessed firsthand… different Service members in different situations, that they don’t have somebody there with them, either mom, dad grandma, girlfriend, whoever it may be, they’re just not prone to heal as fast as somebody with a support system.” *– Speaker from group #4*,* UE Limb Loss group from Bethesda*
Vocational Impact	“My injury has affected my career, as I have not been able to go back to work since my accident. One reason is because I was a purchasing manager and a training manager, and being on the type of medication I was on, I don’t trust myself making fifty thousand dollar purchases and keeping up with spreadsheets. I tried to work from home shortly after the accident, and I looked at some paperwork that I had wrote, and I couldn’t read of understand what I had written… And then the next thing is that I’m in therapy three, four times a week, so how could I possibly work?” *– Speaker from group #7*,* Limb Preservation group from Bethesda*
Independence	“With the loss of an extremity, what makes it so different is now even though we don’t want to, we have to ask for help, because there are things, because we are physically limited, there are things that we can no longer do.” *– Speaker from group #4*,* UE Limb Loss group from Bethesda*
Social Activities	“So, socially, this [orthotic brace] has improved my social life myself, not only with my family but with my peers. I’m able to do more things, go out with them, go hiking… I’m able to do functions with them now.” *– Speaker from group #6*,* UE Limb Loss group from Bethesda*
Loneliness/ Social Isolation	“I feel like I’m on the out-crowd… and I don’t feel like I can talk to anybody.” *– Speaker from group #16*,* Limb Preservation group from San Diego*

Notes: UE = upper extremity; LE = lower extremity

#### Overall results

The child and grandchild codes can also be understood by looking at them holistically, not considering parent domain. Considering the top 10 most frequently used codes, when looking at the entire sample, Resilience was by far the most frequently applied code (10%), followed by Vocational Impact (5%), Relationship with Spouse/Significant Other (5%), Prosthesis (4%), Satisfaction with Physical Abilities/Athleticism (4%), Pain (4%), Anxiety/Fear/PTSD (3%), Medication (3%), Other Relationships (3%), and Anger (3%). See Table 6 for frequency of mention results for any code that represented at least 2% of coded text in at least one participant subgroup (31 codes), with frequency of mention broken out for SMs vs. clinicians, and for the clinical subgroups.

**Table 6:** Most frequently discussed codes across all parent codes (%)

Code	Overall	Participant Group		SMs by Injury Location			SMs by Intervention Type		
		Clinicians	SMs	Upper	Lower	Both	Amputation	Preservation/ Reconstruction	Both
Resilience	**10.2**	**9.3**	**10.3**	**9.3**	**11.5**	**9.3**	**10.1**	**14.4**	**8.8**
Vocational Impact	**5.1**	**6.7**	4.8	2.4	**5.8**	4.7	**5.7**	**5.4**	3.5
Relationship with Spouse/Significant Other	4.9	**6.0**	4.7	**6.3**	4.6	4.2	**5.5**	3.0	4.5
Prosthesis	4.3	**5.9**	4.0	^**§§§^ **12.9**	2.9	1.7	**7.3**	0.3	1.6
Satisfaction with Physical Abilities/Athleticism	4.0	3.7	4.1	3.1	4.5	4.0	3.1	**5.5**	4.8
Pain	3.7	2.1	4.0	1.0	3.4	**6.0**	1.6	4.3	**6.8**
Anxiety/Fear/PTSD	3.2	2.3	3.4	2.0	4.5	2.6	4.5	3.0	2.2
Medication	3.1	1.1	3.4	2.0	3.0	4.5	1.9	**5.5**	4.3
Other Relationships	2.9	2.6	2.9	3.7	2.3	3.3	3.9	1.5	2.4
Anger	2.8	2.5	2.9	4.1	2.2	3.2	3.2	1.5	3.1
Environmental Factors	2.8	3.5	2.7	3.3	3.3	1.7	2.4	3.7	2.6
Grief & Loss	2.7	1.6	2.8	2.1	2.5	3.6	2.4	3.9	3.0
Ambulation	2.6	1.8	2.7	0.4	3.2	3.1	1.5	3.9	3.8
Future Outlook	2.4	2.7	2.3	1.0	3.2	1.7	2.3	3.5	1.9
Amputation/Limb Loss	2.4	^*^ **5.9**	^††^1.8	2.9	2.0	1.0	2.0	1.8	1.5
Self-Esteem	2.3	^*^ **5.5**	^††^1.7	1.4	1.3	2.4	2.1	0.6	1.8
Social Relationships	2.2	2.3	2.2	2.7	2.2	2.0	2.1	1.5	2.6
Family Relationships	2.2	1.5	2.3	0.5	1.9	3.5	1.8	2.0	3.0
Children	2.2	0.8	2.5	1.5	1.7	3.9	1.9	0.3	4.2
Medical Health-Issues	2.1	2.2	2.1	0.7	1.8	2.9	1.9	2.4	2.2
Miscellaneous Emotional Health	1.9	1.9	1.9	1.2	1.7	2.3	2.3	1.3	1.6
Stigma	1.8	1.4	1.9	^*§^3.8	1.6	1.4	2.9	0.4	1.2
Parents	1.8	1.0	1.9	1.9	2.6	1.0	2.3	^*§^4.1	0.4
Positive Affect and Well-being	1.7	0.7	1.9	1.5	1.8	2.2	1.9	1.5	2.1
Independence	1.5	2.6	1.3	1.3	1.4	1.2	1.3	1.2	1.4
Discretionary/Leisure Activities	1.3	1.5	1.3	2.4	1.2	0.8	1.8	0.6	0.8
Orthosis	1.1	0.2	1.3	0.3	1.4	1.5	0.1	^***§§^3.6	1.6
Musculoskeletal	0.9	0.4	1.0	0.6	1.2	1.0	0.4	^**§§^2.7	1.1
Transportation	0.9	0.5	0.9	^*§^2.1	0.7	0.7	1.2	0.5	0.8
Limb Preservation	0.8	^**^2.2	^†††^0.6	0.5	0.6	0.6	0.3	1.0	0.7
Eating	0.7	0.0	^†^0.8	^***§§§^3.2	0.3	0.4	1.2	0.4	0.5

Notes: Values with ≥ 5% of comments for a given column are emphasized in bold.^*^Values that are 2 times the overall frequency;^**^ Values that are 2.5 times the overall frequency;^***^ Values that are 3 times the overall frequency;^†^Values that differ by more than 2 times for clinicians vs. SMs;^††^Values that differ by more than 2.5 times for clinicians vs. SMs;^†††^Values that differ by more than 3 times for clinicians vs. SMs;^§§^Values for clinical subgroups that differ by more than 2.5 times from the overall SM value;^§^Values for clinical subgroups that differ by more than 2 times from the overall SM value;^§§§^Values for clinical subgroups that differ by more than 3 times from the overall SM value

For the most part, SMs and clinicians discussed topics with a similar frequency, with a few exceptions. Clinicians discussed Amputation/Limb Loss and Self-Esteem 2.5 times more frequently than did SMs (6% and 2% for each code, respectively). Similarly, clinicians discussed Limb Preservation three times more frequently than did SMs (2% and < 1%, respectively). In contrast, SMs were the only ones to discuss Eating; this topic was never mentioned in the clinician groups. There were also some differences observed among clinical subgroups. Compared to the overall rate of discussion for SMs in general, participants with upper extremity injuries/loss discuss Stigma and Transportation twice as often, and Prosthesis and Eating three times as often. SMs with limb preservation/reconstruction discussed Parents twice as often, and Orthosis and Musculoskeletal 2.5 more often compared to SM participants overall. These comparisons should be interpreted cautiously given the limitations of drawing inferences from this type of qualitative data. See Table 6 for notation on these comparisons. Table 7 contains exemplar quotes for the codes more frequently discussed by select clinical groups.

**Table 7:** Exemplar quotes of most frequently discussed codes for SMs by clinical subgroup

Code	Clinical Subgroup with Highest Frequency	Exemplar Quote
Prosthesis	Upper Extremity Injury/ Limb Loss	“I used to do a lot of cooking, and now there’s just times where you just, oh my God, I swear to God, I’m going to stab someone. You know, going from being able to do something as simple as hold the damn tomato and slice it to now when I try to hold a tomato, I crush it, you know. Because I can’t hold a knife in my prosthetic, so then when I try and stabilize it with my prosthetic, which is awesome until you get to smushy things, like tomatoes and things like that. Or trying to hold a bottle of Coke, or a bottle of water, because it’s hot outside, and my arm is sweaty. My hand has decided to close without me telling it to. Now I’m wearing the entire bottle of water.” *– Speaker from group #6*,* UE Limb Loss group from Bethesda*
Stigma	Upper Extremity Injury/ Limb Loss	“I think that you’re always getting stares. People are staring. I don’t like to be stared at. And then I’ve probably explained how this thing works thousands of times to people. I mean, I understand that people have questions, and they want to know how it works, and they want to know what happened, and – but for me, just talking about it over and over again kind of gets… really old.” *– Speaker from group #10*,* UE and LE group from San Antonio (comprised of individuals with limb loss and/or limb preservation)*
Transportation	Upper Extremity Injury/ Limb Loss	“So, I would say that the biggest change is just you can’t go out and just do anything. Everything’s got to have some forethought to it. I mean, I can’t even just go and buy a car anymore. It’s, well, okay, where are the controls located? How am I going to operate a turn signal? How am I going to turn on my freaking windshield wipers? Where’s the radio located? Where’s whatever sort of shift mechanism is in the vehicle? Where’s that located? Where are my mirrors located? Can I operate those easily, if I need to change them while driving? It’s just – it’s everything.” *– Speaker from group #6*,* UE Limb Loss group from Bethesda*
Eating	Upper Extremity Injury/ Limb Loss	“You’re hesitant to go out and eat on your own, and you have to be careful of what you eat. So typically, you can’t order steak, or I don’t. Because of the cutting part. When I order, I always try to order things I can handle on my own.” *– Speaker from group #6*,* UE Limb Loss group from Bethesda*
Parents	Limb Preservation/ Reconstruction	“I wouldn’t consider myself an angry person but I have lots of anger outbursts. It wasn’t just – It was a combination of all the things. And I would just take that out in a second on strangers or especially people that you’re close to I found. Sometimes – Like my mom came and stayed with me after things fell apart at home with my career and everything. She stayed here a total of about eight months with me. I mean – And she’s the one who helped me out more than anyone and I would just be a real ass.” *– Speaker from group #1*,* LE Preservation group from Bethesda*
Orthosis	Limb Preservation/ Reconstruction	“You may try like nine things that don’t work and get the one thing that does. There’s a process. I spent five hours yesterday trying on a new harness that I took home today, and absolutely the most uncomfortable, terrible thing I’ve – but people have got to try different things. So, it definitely is a process.” *– Speaker from group #6*,* Limb Preservation group from San Antonio*
Musculoskeletal	Limb Preservation/ Reconstruction	“I had ankle fractures… and multiple fractures in the feet. I was able to return to duty on a limited status. I was able to powerwalk and lived for almost five years with just decreasing function and increasing pain but redeployed to Afghanistan several years later by being able to powerwalk. Then arthritis got to the point where things were so bad, I was considering an elective amputation of both feet because I was scared to fuse. Cases of single fusions, there are a lot of them in the military. But there are currently more single and double amputees than there are people with bilateral fusions that have chosen to stay in due to limitations with bracing technology.” *– Speaker from group #1*,* LE Preservation group from Bethesda*

Notes: UE = upper extremity; LE = lower extremity

## Discussion

Trauma is unique from other causes of limb loss, which are often associated with advanced age and poor health [40]. The individuals who experience major extremity trauma are typically young, healthy, and physically active when a sudden injury changes the course of their lives irreversibly. Previous research has documented the experiences of recovery, rehabilitation, and pain after major orthopedic trauma [60–64]. This study is unique, however, in that we have comprehensively identified and enumerated the domains of functioning and factors relevant to HRQOL for this population, in a format conducive to developing PRO measures [20, 42–44].

Participants described issues of physical and emotional health as well as social participation that are measured by existing high-quality PRO measures relevant to the general population (e.g., pain, fatigue, depression, loneliness [65–68]) and for people with other physical injuries and disabilities (e.g., resilience, fine-motor function, self-care [59, 69–71]), but they also identified several unique factors. These included satisfaction with prosthetic and/or orthotic devices, satisfaction with physical abilities and athleticism, body image, future outlook, and the vocational impacts of injury. Similar themes have been identified in other recent qualitative research with combat-related [72, 73], civilian [74, 75], and international [62, 76, 77] individuals with limb loss. Development of new PRO measures to assess these topics will be useful, and is underway [78].

This study was unique in considering and including the perspectives of a broad array of manifestations of traumatic injuries, by including individuals with upper- and lower-extremity injuries, as well as those with limb loss and limb preservation/reconstruction. While most orthopedic research focuses on one injury (i.e., specific injury type or location) or intervention (i.e., one surgical technique or device), this project included a diverse group of patients, including those with *multiple* injuries. Some injury causes (e.g., combat blast exposure, motor vehicle accidents) can affect more than one limb; we suspect that the experiences of these patients are often overlooked in narrowly framed research in the field of orthopedics.

Unsurprisingly, experiences with device use are important for these individuals’ HRQOL. Prostheses were more commonly discussed by those with upper-limb injuries, which may reflect the complexity of fine motor tasks, challenges of completing these with an external device, and advancements in device technology [79–82]. Orthotic devices were discussed by those with limb preservation/reconstruction, which matches the target audience for many available devices [83, 84]. Stigma was brought up frequently by those with upper-extremity injuries, likely given the more visible nature of these injuries, which are less easily hidden with clothing [85]. While the comparisons noted in this manuscript are based on descriptive information from qualitative data and cannot be used to make strong inferences, we believe they are useful for shaping PRO development to be inclusive of the diverse experiences of major extremity trauma, and grounding the results in the perspectives of the participants’ lived experiences.

### Study limitations

The primary limitation of this research relates to the sample, which was one of convenience. The SMs were recruited from the population served by the participating centers. Therefore, the SMs involved in this study may have had unusual access to adaptive sports, advanced prostheses, and intensive rehabilitation, which may be rare in the broader community in the U.S. or globally. There were two issues with provider data collection: one session included only one participant due to scheduling challenges, and demographic information was not provided by the recruitment sites for 16 participants. Although we want to acknowledge these as potential limitations, we do not believe either has a meaningful impact on the interpretability or generalizability of these results. However, one limitation to transferability of results is that all participants with lived experience of limb trauma were SMs—who are known to have access to services and supports (both instrumental and cultural) not available to civilians [64, 80, 86]—and were primarily male. It would be beneficial to replicate the results in a civilian population, and to include more females in future data collection. Additionally, it would be beneficial to conduct repeat interviews with a subset of the original focus group participants to provide feedback on our findings.

## Conclusions

SMs and their clinical providers generated rich content about HRQOL after major extremity trauma. This investigation revealed similar HRQOL topics as identified as important in other populations and covered by existing PRO measures. These might apply to extremity trauma populations, but content validity would need to be confirmed. Furthermore, these results can inform the creation of new PRO measures tailored for this population, including satisfaction with orthosis/prosthesis, satisfaction with physical abilities/athleticism, body image, future outlook, and vocational impact.

## Electronic supplementary material

Below is the link to the electronic supplementary material.

Supplementary Material 1

Supplementary Material 2
