# Supplementary material for: Health-related quality of life after major extremity trauma: qualitative research with military service members and clinicians to inform measurement of patient-reported outcomes
Source: Qual Life Res. 2025 Feb 20;34(12):3351–64. doi: 10.1007/s11136-025-03915-0 (PMC12689715; doi:10.1007/s11136-025-03915-0)
Supplement: Supplementary file 2 — Supplementary Material 2 [file 11136_2025_3915_MOESM2_ESM.docx]

**Supplementary Material**

**Article Title:** Health-related quality of life after major extremity trauma: Qualitative research with military service members and clinicians to inform measurement of patient-reported outcomes

**Journal:** *Journal of Quality of Life Research*

**Author Names:** Tyner, C.E., Kisala, P.A., Slotkin, J., Cohen, M.L., Cancio, J.M., Pruziner, A.L., Dearth, C.L., & Tulsky, D.S.*

**Affiliation and email for corresponding author*:** Center for Health Assessment Research and Translation, Departments of Physical Therapy and Psychological & Brain Sciences, University of Delaware, Newark, DE, USA; dtulsky@udel.edu

## Supplemental Details on Participants and Focus Group Design

Focus group participants were recruited from three Military Treatment Facilities (Walter Reed National Military Medical Center in Bethesda, MD; Brooke Army Medical Center in San Antonio, TX; and Naval Medical Center San Diego in San Diego, CA) and one Department of Veterans Affairs (VA) medical center (James A. Haley VA Medical Center in Tampa, FL). The groups were held at the participating medical centers with the exception of the San Diego groups which were held at a rented, off-site conference room. Participants included SMs with medically documented upper and/or lower extremity loss and/or preservation/reconstruction and clinical providers with at least 2 years of experience with limb trauma and loss. All participants with lived experience needed to be at least 18 years of age at the time of study participation and needed to demonstrate the cognitive capacity to provide informed consent. Attendance at groups was limited to the researchers and participants. All research activities were approved by the lead site (University of Delaware) IRB and by the local institutional review board at each participating facility.

A convenience sample of participants were recruited locally from lists of current and former patients at each participating site. Site investigators and local study personnel used a variety of IRB-approved recruitment methods (e.g., face-to-face, phone, email) and were instructed to recruit for 3 groups per site, with a goal of 4-6 participants per group. Investigators were further instructed to recruit a diverse group of individuals with regard to limb(s) affected (upper vs. lower vs. both) and treatment (amputation vs. limb preservation). Since the majority of potential participants were individuals with lower limb loss, specific emphasis was placed on recruiting individuals with upper extremity injuries as well as on recruiting individuals who underwent limb preservation procedures on at least one limb. Four sites hosted a separate focus group with clinicians. Sites did not report on refusal rates, and the study design did not allow for participants to review transcripts or participate in repeat interviews.

Five PhD-level psychologists who worked as researchers served as focus group moderators (DT, DV, MLC, HB, and KM). Each group was facilitated by two trained, PhD-level moderators. At least one of the project investigators (DT or DV) served as the lead moderator for every group, and groups were co-moderated by DT, DV, HB, KM, or MLC depending on availability. None of the moderators participated directly in the data analysis, although DT helped to interpret the final results. None of the moderators have lived experience of major extremity trauma, and none were known to the participants. The moderators were introduced to the participants as researchers interested in improving measurement of quality of life after limb trauma. The moderators’ role was to introduce the activity, encourage participation from each group member, and provide an organized structure to the discussion. The moderators’ primary goal was to foster a spontaneous discussion, with limited prompting or directing. In each group, the discussion began with open-ended questions about HRQOL after limb trauma, for example, “how is your life affected by your traumatic injury or limb loss?” and “What do you think of when I say the phrase ‘quality of life’ as it relates to your health?” The moderators were interested in covering most major domains of HRQOL (physical, emotional, and social), but did not press if a domain did not generate much conversation. Prompts were rephrased for clinician groups to ask about their clinical and professional experiences. Each focus group lasted between 60 to 90 minutes. This methodology parallels that used in the development of PROMIS and Neuro-QoL instruments and was selected to capture stakeholder feedback as thoroughly as possible while attempting to minimize the effects of researcher bias.

## Table S1. Frequency of Child Codes Under the Physical Health Parent Code (%)

|  |  | **Participant Group** | | **SMs by Injury Location** | | | **SMs by Intervention Type** | | |
| --- | --- | --- | --- | --- | --- | --- | --- | --- | --- |
| **Child Code** | **Overall** | **Clinicians** | **SMs** | **Upper** | **Lower** | **Both** | **Limb Loss** | **Preservation/ Reconstruction** | **Both** |
| Medical Health/Issues^*^ | 26.7 | 43.7 | 23.9 | 22.3 | 26.7 | 21.4 | 25.3 | 32.0 | 19.0 |
| Mobility | 15.7 | 10.5 | 16.6 | 8.5 | 18.6 | 17.4 | 15.0 | 13.3 | 19.4 |
| Orthosis/Prosthesis | 15.1 | 17.0 | 14.8 | 37.6 | 12.2 | 8.7 | 25.5 | 9.5 | 7.7 |
| Pain | 12.1 | 6.8 | 13.0 | 3.1 | 11.9 | 18.1 | 7.3 | 10.7 | 19.0 |
| Satisfaction with Physical Abilities/Athleticism | 11.1 | 10.2 | 11.3 | 8.7 | 12.8 | 10.6 | 10.4 | 12.9 | 11.4 |
| Medication | 9.6 | 3.7 | 10.6 | 5.9 | 10.0 | 13.2 | 6.9 | 16.1 | 11.4 |
| Upper Extremity Function/ Self Care | 6.0 | 3.0 | 6.5 | 13.6 | 4.4 | 6.0 | 7.2 | 3.1 | 7.3 |
| Sleep Disturbance | 1.9 | 1.2 | 2.0 | 0.0 | 1.0 | 3.8 | 1.2 | 0.0 | 3.5 |
| Fatigue | 0.4 | 1.8 | 0.1 | 0.0 | 0.1 | 0.1 | 0.1 | 0.1 | 0.1 |
| Sexual Health | 0.4 | 0.9 | 0.3 | 0.2 | 0.5 | 0.0 | 0.2 | 0.1 | 0.3 |
| Miscellaneous Physical Health | 1.1 | 1.2 | 1.0 | 0.2 | 1.7 | 0.6 | 0.8 | 2.2 | 0.7 |

*Note:* ^*^Medical Health/Issues included the grandchild codes of Cardiovascular, Genitourinary, Musculoskeletal, Orthopedic Surgery, Peripheral Nervous System, Skin Issues, and Medical Treatments/Therapies. Great-grandchild codes under Musculoskeletal were Arthrosis/Arthritis, Contractures, Fractures, Heterotopic Ossification, Muscle Atonia, Osteomyelitis, Residual Limb, Soft Tissue Infection, and Spasticity. Great-grandchild codes under Peripheral Nervous System were Loss of Sensation, Neuromas, and Paralysis.

## Table S2. Frequency of Child Codes Under the Emotional Health Parent Code (%)

|  |  | **Participant Group** | | **SMs by Injury Location** | | | **SMs by Intervention Type** | | |
| --- | --- | --- | --- | --- | --- | --- | --- | --- | --- |
| **Child/Grandchild^*^ Code** | **Overall** | **Clinicians** | **SMs** | **Upper** | **Lower** | **Both** | **Limb Loss** | **Preservation/ Reconstruction** | **Both** |
| Resilience | 32.5 | 30.6 | 32.8 | 32.7 | 35.5 | 29.5 | 29.3 | 46.1 | 31.7 |
| Anxiety/Fear/PTSD | 10.3 | 7.7 | 10.7 | 6.9 | 13.9 | 8.2 | 12.9 | 9.8 | 7.8 |
| Anger | 9.0 | 8.3 | 9.1 | 14.4 | 6.6 | 10.2 | 9.3 | 4.7 | 11.1 |
| Grief/Loss | 8.5 | 5.2 | 9.0 | 7.3 | 7.6 | 11.5 | 6.9 | 12.6 | 10.6 |
| Future Outlook | 7.6 | 8.9 | 7.4 | 3.7 | 9.9 | 5.6 | 6.5 | 11.2 | 6.7 |
| Self-Esteem | 7.3 | 18.0 | 5.5 | 5.0 | 4.1 | 7.6 | 6.2 | 1.8 | 6.3 |
| Body Image^*^ | 3.7 | 4.6 | 3.5 | 2.5 | 2.7 | 5.0 | 4.0 | 0.0 | 4.6 |
| Stigma | 5.7 | 4.6 | 5.9 | 13.3 | 4.8 | 4.5 | 8.4 | 1.2 | 4.3 |
| Positive Affect and Well-being | 5.5 | 2.3 | 6.0 | 5.3 | 5.4 | 7.0 | 5.5 | 4.7 | 7.5 |
| Depression | 3.8 | 2.9 | 3.9 | 3.9 | 4.3 | 3.4 | 4.0 | 3.9 | 3.8 |
| Health-Related Self Efficacy | 0.2 | 0.8 | 0.1 | 0.9 | 0.0 | 0.0 | 0.3 | 0.0 | 0.0 |
| Miscellaneous Emotional Health | 6.0 | 6.2 | 5.9 | 4.1 | 5.3 | 7.4 | 6.7 | 4.1 | 5.6 |

## Table S3. Frequency of Child Codes Under the Social Participation Parent Code (%)

|  |  | **Participant Group** | | **SMs by Injury Location** | | | **SMs by Intervention Type** | | |
| --- | --- | --- | --- | --- | --- | --- | --- | --- | --- |
| **Child Code** | **Overall** | **Clinicians** | **SMs** | **Upper** | **Lower** | **Both** | **Limb Loss** | **Preservation/ Reconstruction** | **Both** |
| Social Relationships^*^ | 63.6 | 53.0 | 65.3 | 64.9 | 63.3 | 68.0 | 64.0 | 64.0 | 68.0 |
| Vocational Impact | 18.0 | 23.6 | 17.1 | 8.0 | 20.7 | 16.9 | 17.6 | 24.7 | 13.4 |
| Independence | 8.9 | 10.2 | 8.6 | 9.1 | 8.2 | 8.9 | 6.7 | 7.8 | 11.9 |
| Social Activities | 7.7 | 9.3 | 7.5 | 14.7 | 7.3 | 4.4 | 10.1 | 2.9 | 5.1 |
| Loneliness/Social Isolation | 1.6 | 3.1 | 1.4 | 3.2 | 0.4 | 1.7 | 1.5 | 0.6 | 1.4 |
| Miscellaneous Social Participation | 0.2 | 0.9 | 0.1 | 0.0 | 0.1 | 0.2 | 0.0 | 0.0 | 0.3 |

*Note:* ^*^Social Relationships was comprised of the grandchild codes Family Relationships (including the great-grandchild codes of Children, Parents, and Spouse/Significant Other), Friend Relationships, and Other Relationships
